# Case report: Oxaliplatin-induced idiopathic non-cirrhotic portal hypertension: a case report and literature review

**DOI:** 10.3389/fmed.2023.1285064

**Published:** 2023-11-28

**Authors:** Jiayuan Ye, Yilian Xie, Yaojiang Xu, Nan Chen, Yifei Tu

**Affiliations:** ^1^Department of Infectious Diseases, Shangyu People's Hospital of Shaoxing, Shaoxing, Zhejiang, China; ^2^Department of Infectious Diseases, The First Affiliated Hospital of Ningbo University, Ningbo, Zhejiang, China; ^3^Department of Radiology, Shangyu People's Hospital of Shaoxing, Shaoxing, Zhejiang, China

**Keywords:** oxaliplatin, variceal banding, idiopathic non-cirrhotic portal hypertension, gastrointestinal tumors, case report

## Abstract

Oxaliplatin has become a widely used agent in neoadjuvant chemotherapy for gastrointestinal tract tumors and is an integral part of the therapeutic approach for managing colorectal cancer recurrences and metastases, resulting in a more favorable prognosis for patients. Nevertheless, oxaliplatin can give rise to idiopathic non-cirrhotic portal hypertension (INCPH). The emergence of INCPH can disrupt tumor chemotherapy and incite persistent adverse reactions in later stages, significantly complicating clinical management. Consequently, we have presented a case report of INCPH induced by oxaliplatin chemotherapy with the aim of advancing the diagnosis and treatment of this condition, with a particular focus on the clinical manifestations. This study has ascertained that the condition is primarily attributed to complications related to portal hypertension, such as gastrointestinal bleeding, splenomegaly, and hypersplenism. The pathological features primarily involve hepatic sinus dilation and congestion, portal obstruction, absence, stenosis, shunting, localized venous and perisinusoidal fibrosis, as well as hepatocellular atrophy. Treatment primarily concentrates on strategies typically employed for cirrhosis. Endoscopic ligation, sclerotherapy, and non-selective beta-blockers (NSBBs) can be selected to prevent and treat variceal hemorrhage. Transjugular intrahepatic portosystemic shunt (TIPS) and liver transplantation can also be chosen for severe cases. Notably, despite the timely discontinuation of oxaliplatin, most patients continue to experience disease progression, ultimately resulting in a poor prognosis due to either tumor advancement or the ongoing progression of portal hypertension. This emphasizes the importance for physicians to be aware of and consider the risk of INCPH when prescribing oxaliplatin.

## Introduction

Idiopathic non-cirrhotic portal hypertension (INCPH) is a global phenomenon, with notably higher incidence rates in developing countries compared to Western European nations. The prevalence is significantly elevated in Asia, particularly in India and Japan. INCPH accounts for approximately 25% of patients presenting with portal hypertension in India (1, 2), whereas the incidence of INCPH has dramatically decreased to just 11 new cases annually in Japan (3). In North America and Europe, INCPH is regarded as a rare condition, constituting only 3–5% of portal hypertension cases (4).

The diagnosis of INCPH is primarily exclusionary, requiring adherence to existing diagnostic criteria and the exclusion of other causes of portal hypertension and liver diseases (5, 6): (1) the presence of clear manifestations of portal hypertension, (2) the exclusion of chronic liver diseases, such as chronic viral hepatitis, fatty liver, alcoholic liver disease, autoimmune hepatitis, primary biliary cirrhosis (PBC), and Budd–Chiari syndrome, which can lead to portal hypertension based on appropriate serological tests and liver biopsies, and (3) radiological examinations demonstrating the patency of the portal and hepatic veins.

Oxaliplatin is most commonly associated with sinusoidal vein obstruction syndrome (SOS), characterized by liver enlargement, stiffness, pain, ascites, and jaundice. Severe cases can present with multiple organ failure or encephalopathy and other central nervous system abnormalities (7). In contrast, the incidence of INCPH induced by oxaliplatin is relatively low, with the main clinical features being portal hypertension, splenomegaly or giant splenomegaly, and hypersplenism. Liver injury, ascites, and hepatic encephalopathy are less common.

In this study, we report a case of a 56-year-old male who developed progressive portal hypertension after completion of oxaliplatin chemotherapy follow-up examinations and was finally diagnosed with oxaliplatin-induced INCPH. We aim to alert healthcare providers using oxaliplatin to be vigilant for such rare complications and to be aware of the persistent damage that can occur even after discontinuing oxaliplatin treatment.

## Case report

The affected individual, a 58-year-old male residing in China, was diagnosed with gastric cancer during a routine physical examination in March 2018. The pathological analysis revealed signet cell carcinoma of the stomach. Prior to surgical intervention, the patient underwent four cycles of chemotherapy using the SOX regimen (tegafur: 60 mg bid d1-14; oxaliplatin: 240 mg d1 q3W). The surgical procedure involved “radical gastrectomy (Billroth II+ BRAUN) + D2 lymph node dissection + cholecystectomy.” Following surgery, the patient continued chemotherapy with SOX for four additional cycles, and in the last three cycles, the oxaliplatin dose was reduced (tegafur: 60 mg bid d1-14; oxaliplatin: 230 mg d1 q3W) to complete the total eight-cycle treatment, with a cumulative oxaliplatin intake of 1,890 mg. In October 2019, the patient underwent a routine full-abdomen scan following the completion of chemotherapy, which revealed portal hypertension. As part of the preliminary etiological screening, a hepatitis B series of tests was performed, which indicated a mild elevation in hepatitis B surface antigen (HBsAg), with a value of 1.77 COI. Hepatitis B DNA was undetectable at that time, and antiviral treatment was not initiated. In July 2020, HBsAg levels quickly decreased to 1.08 COI. By December 2020, during a follow-up examination, HBsAg had already turned negative (Table 1).

**Table 1:** Patient's flowchart.

**Events**	**Surgical treatment**	**Portal hypertension**	**Carvedilol** + **Routine follow-up**		**Hospitalization + Liver biopsy**	**Routine follow-up**
**Time**	**D0**	**D**+**1 year**	**D**+**2 years**		**D**+**3 years**	**D**+**4 years**
**Found gastric cancer**						
**The laboratory examination of hepatitis B**.						
Project	2018	2019	2020.7	2020.12	2021	Reference range
HBsAg (COI)	0.9990	1.7700	1.0800	0.9400	0.7500	0.000–1.000
HBsAb (IU/L)	< 2.0	< 2.0	< 2.0	< 2.0	< 2.0	0.000–1.000
HBeAg (COI)	0.0830	0.0700	0.0700	0.0900	0.0700	0.000–1.000
HbeAb (COI)	0.0050	0.0000	0.0000	0.0000	0.0100	>1.000
HBcAb (COI)	0.0080	0.0100	0.0100	0.0100	0.0100	>1.000
HBV DNA (IU/ml)	BDL	BDL	BDL	BDL	BDL	< 30

**Figure 1:**
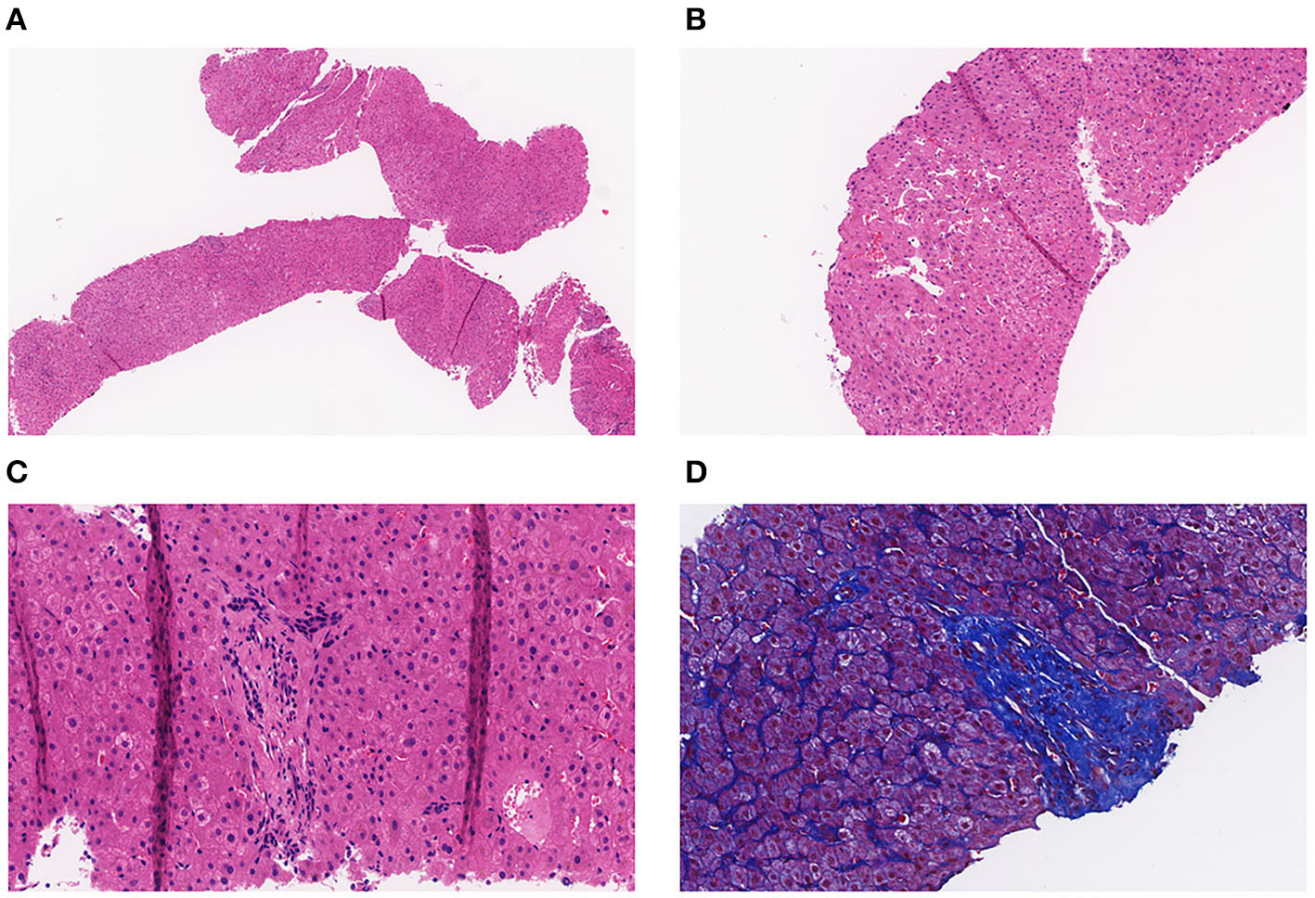
**(A)** Pathology of the liver tissue: HE staining (X40). **(B)** Pathology of the liver tissue: HE staining (X100). **(C)** Pathology of the liver tissue: HE staining (X200). **(D)** Pathology of the liver tissue: MASSON staining (X200).

A full-abdomen routine enhanced scan was performed in June 2021, revealing a slight enlargement of the left liver lobe, a slightly increased interlobular space, and an intact capsule. No signs of bile duct dilation, either intrahepatic or extrahepatic, were observed, and the portal vein measured approximately 17 mm in width. Mild dilation of the splenic vein and superior mesenteric vein was noted, along with splenomegaly. Due to unexplained portal hypertension, the patient was admitted to Shaoxing Shangyu People's Hospital for further evaluation and treatment in August 2021. The physical examination, CBC, biochemical, and immune indicators of the patient on admission are shown in Table 2. On 18 August 2021, portal vessel computed tomography (CT) angiography demonstrated unobstructed blood flow, indicating the presence of portal hypertension and splenomegaly. Subsequently, a liver biopsy performed on 25 August 2021 revealed evidence of chronic liver damage (G1S1), characterized by vague nodular regeneration and focal fibrosis. The biopsy also indicated partial hepatocyte atrophy and reduced portal veins in transitional regions, along with fibrous tissue hyperplasia (Figure 1). After a comprehensive assessment, the patient met all three diagnostic criteria for INCPH and was ultimately diagnosed with oxaliplatin-induced INCPH.

**Table 2:** Patient's physical and laboratory examination.

**Tests**	**Result**	**Reference range**	**Interpretation**
**Patient's vitals**			
BP (mmHg)	100/68	< 120/80	Normal
HR (beats/min)	75	60–100	Normal
RR (breaths/min)	18	20-Dec	Normal
T (°C)	36.7	37	Normal
**CBC test**			
WBC (10^*^9/L)	3	3.5–9.5	Low
NEU (10^*^9/L)	2.19	1.8–6.3	Normal
EOS (10^*^9/L)	0.1	0.02–0.52	Normal
LYM (10^*^9/L)	0.59	1.1–3.2	Low
RBC (10^*^9/L)	4.06	4.3–5.8	Normal
HGB (g/l)	117	130–175	Normal
MCV (fl)	88.7	82–100	Normal
MCH (pg)	28.7	27–34	Normal
MCHC (g/l)	324	316–354	Normal
PLT (10^*^9/L)	82	125–350	Low
**Laboratory chemistry**			
ALT (U/L)	23.1	Sep-50	Normal
AST (U/L)	30.6	15–40	Normal
GGT (U/L)	21.9	Oct-60	Normal
ALP (U/L)	74.5	45–125	Normal
TBIL (μmol/L)	26.5	4.5–22	High
DBIL (μmol/L)	8.4	0–6	High
IBIL (μmol/L)	18.1	1.5–14	High
**Immune indicators**			
anti-HAV IgM	-		Negative
anti-HCV IgG	-		Negative
anti-HDV IgG	-		Negative
anti-HEV IgM	-		Negative
anti-HGV IgG	-		Negative
HBsAg (COI)	0.75	0–1.0	Low
HBsAb (IU/L)	< 2	0–10	Low
HBeAg (COI)	0.07	0–1.0	Low
HbeAb (COI)	0.01	>1.0	Low
HBcAb (COI)	0.01	>1.0	Low
CMV DNA (copies)	BDL	400	Negative
CMV IgM	3.18	< 20	Negative
EBV DNA (copies)	BDL	400	Negative
EBV IgM	-		Negative
ANA	Negative	< 1:100	Negative
AMA	-		Negative
AMA-M2	-		Negative
ASMA	-		Negative
LKM-1	-		Negative
LC-1	-		Negative
SLAILP	-		Negative
CER (g/l)	0.25	0.15–0.30	Normal
Fecal eggs	-		Normal

BP, blood pressure; HR, heart rate; RR, respiration rate; T, temperature; WBC, white blood cells; NEU, neutrophil; EOS, eosinophil; LYM, lymphocyte; RBC, red blood cells; HGB, hemoglobin; MCV, mean corpuscular volume; MCH, mean corpuscular hemoglobin; MCHC, mean corpuscular hemoglobin concentration; PLT, platelet; ALT, alanine aminotransferase; AST, aspartate aminotransferase; GGT, gamma-glutamyl transferase; ALP, alkaline phosphatase; TBIL, total bilirubin; DBIL, direct bilirubin; IBIL, indirect bilirubin; ANA, antinuclear antibodies; AMA, anti-mitochondrial antibody; AMA-M2, anti-mitochondrial antibody type II; ASMA, anti-smooth muscle antibody; LKM-1, anti-liver and kidney microsome-1; LC-1, anti-hepatocellular solute-1; SLAILP, anti-soluble liver antigen/liver–pancreas antigen antibody; CER, ceruloplasmin; BDL, below detection limit.

**Figure 2:**
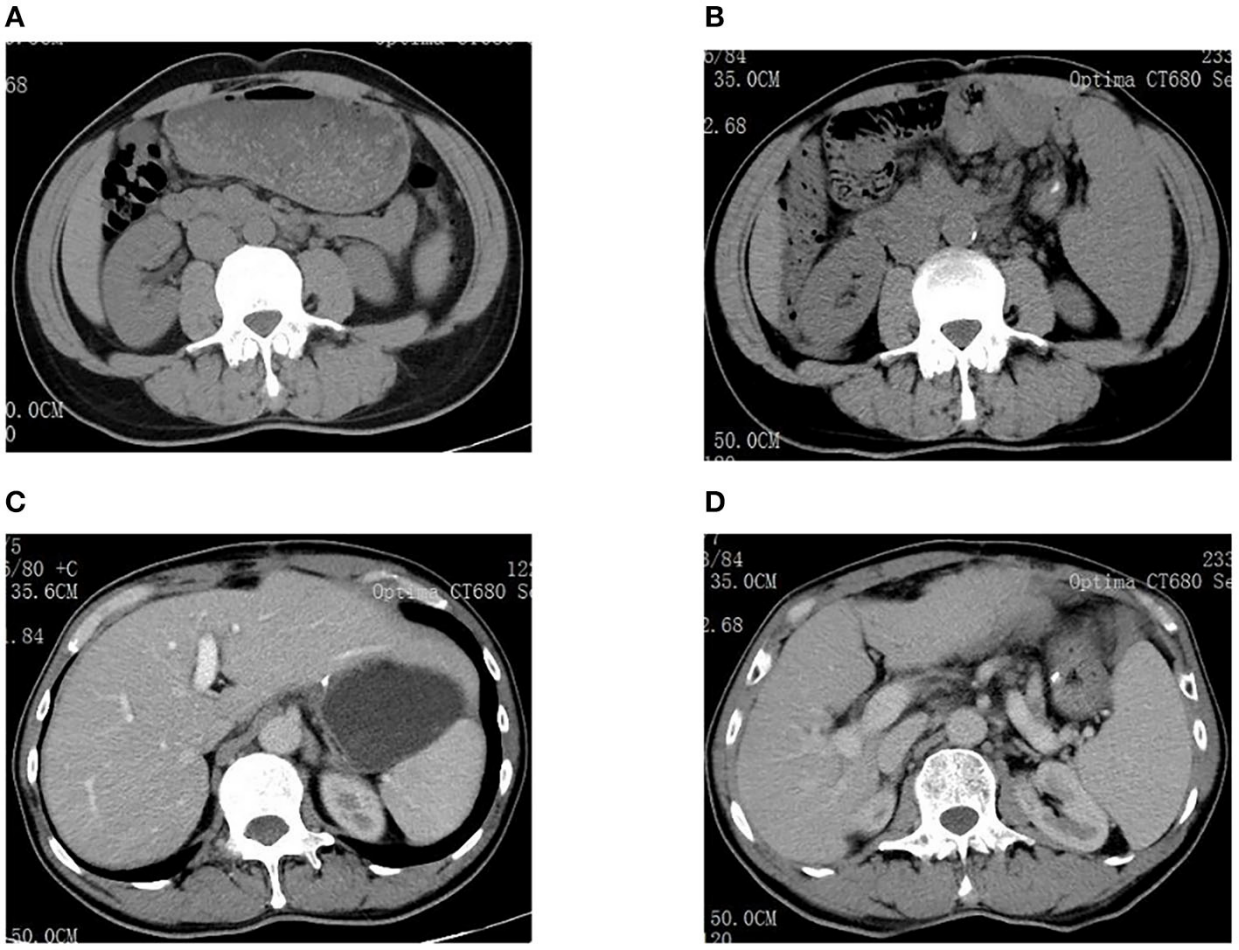
**(A)** Spleen size was normal before surgery in 2018. **(B)** The spleen was significantly enlarged in 2022. **(C)** Liver size was normal before surgery in 2018. **(D)** The portal vein dilated significantly in 2022.

**Figure 3:**
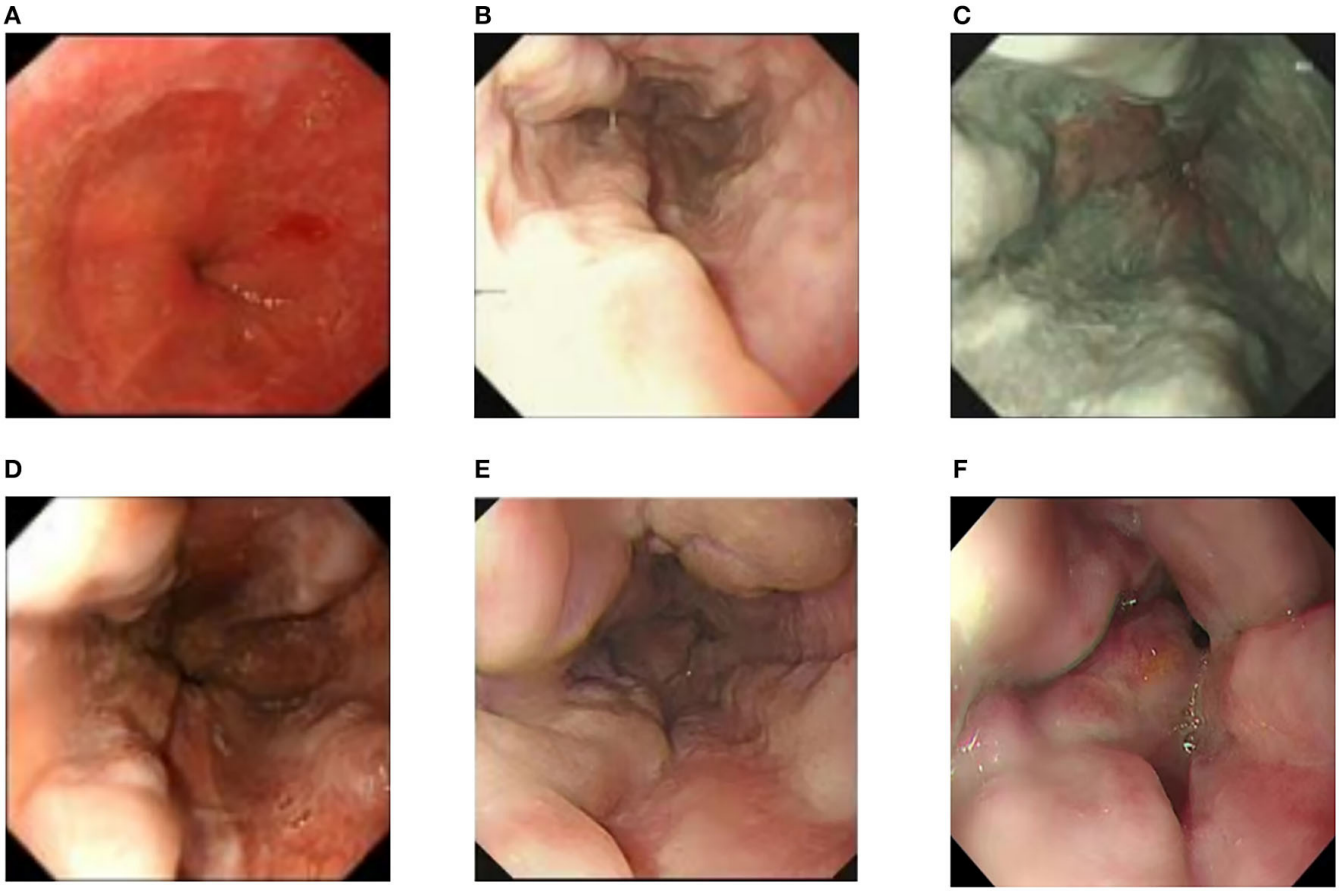
**(A)** Preoperative esophagogastroduodenoscopy showed no significant esophageal or gastric fundal varices. **(B–F)** Annual routine check-ups from 2019 to 2023 showed progressive aggravation of esophageal and gastric fundal varices.

**Figure 4:**
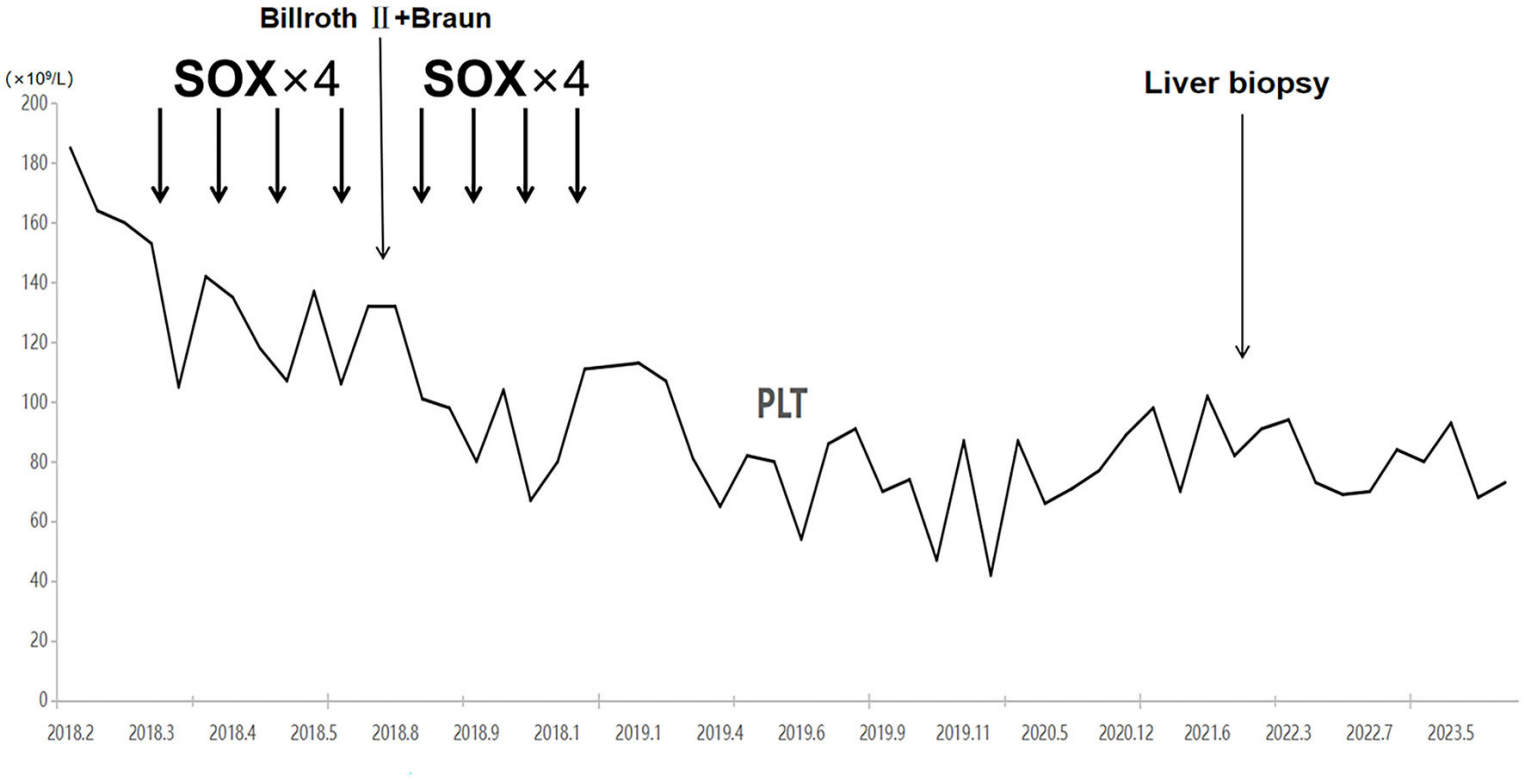
Platelet counts started to decrease with the initiation of chemotherapy and have not recovered to date.

Examinations of the patient conducted over the past 5 years have revealed significant changes when compared to the full-abdomen routine scan performed before the surgery in 2018 and post-surgery in 2022 (Figure 2). Notably, the portal vein has markedly widened, and the spleen has enlarged substantially. A gastroscopy conducted before the surgery in 2018 indicated the absence of significant esophageal and gastric varices. However, subsequent annual routine examinations from 2019 to 2023 have shown a progressive worsening of esophageal and gastric varices (Figure 3). The patient initiated carvedilol (6.25 mg *peros quaque die*) therapy in 2020, but the disease continued to advance. Platelet count, which began to decline after chemotherapy, has not yet returned to normal levels (Figure 4). A PubMed literature search unveiled numerous documented cases of INCPH induced by oxaliplatin.

### Literature review

Using the PubMed search system, a total of 35 relevant articles were retrieved and screened using the keywords “oxaliplatin” and “portal hypertension.” Among these, 21 articles provided case reports, yielding a total of 36 cases for analysis and summarization. Several other articles with relevant cases could not be analyzed due to the unavailability of full texts.

Among the 36 cases identified in the literature, the age of onset ranged from 35 to 81 years, with 17 males and 19 females. The minimum reported course of oxaliplatin treatment leading to the condition was 3 cycles, while the maximum was 37 cycles, with a median of 11 cycles. However, the exact cumulative dosage of oxaliplatin could not be calculated due to incomplete data. Nonetheless, based on available detailed dosage records (estimated body surface area at 1.7 m^2^ for males and 1.5 m^2^ for females), cumulative doses ranged from 540 mg to 3,300 mg, with a median concentration of approximately 1,600 mg. The time of onset of symptoms varied significantly, with some cases experiencing symptoms as early as 3 months after treatment, while others took up to 6 years though the majority appeared within 1–3 years.

Nineteen cases presented with complications of esophagogastric variceal bleeding at the time of diagnosis, and almost all patients exhibited thrombocytopenia and splenomegaly. Imaging findings included esophageal varices on post-chemotherapy endoscopy, and conventional CT and magnetic resonance imaging (MRI) indicated portal hypertension and splenomegaly, with some cases showing liver regenerative nodules. Twenty-four patients underwent liver biopsy, with pathological features primarily including sinusoidal dilation, congestion, portal vein occlusion, absence, stenosis, shunting, localized venous and sinus peri-fibrosis, hepatocellular atrophy, and abnormal vascular aggregation around the central vein.

Regarding treatment, 14 patients underwent surgical procedures involving ligation and local sclerosis, supplemented with PPI and symptomatic treatment. Two patients underwent TIPS treatment, and one patient underwent liver transplantation. Some patients with milder varices chose to lower portal vein pressure. However, in terms of prognosis, it appears that even after discontinuing oxaliplatin, nine patients experienced continued progression of portal hypertension and esophagogastric varices, seven patients had irreversible complications due to tumor progression, resulting in poor outcomes, and the prognosis for nine patients was unclear due to the lack of detailed descriptions. In six cases, follow-up information did not specify whether the progression was related to the primary disease or complications.

## Discussion

Oxaliplatin, a third-generation platinum-based anticancer drug, exerts its antitumor effects by forming cross-links with DNA bases, thereby inhibiting DNA and protein synthesis. It is primarily employed in the treatment of digestive tract tumors (8). The neoadjuvant chemotherapy regimen known as SOX has shown efficacy in the management of locally advanced gastric adenocarcinoma following D2 gastrectomy. This regimen comprises the oral administration of tegafur (S-1) and intravenous delivery of oxaliplatin. The common side effects associated with oxaliplatin encompass neurotoxicity, gastrointestinal reactions, and hematological toxicity. There have been reports from surgeons regarding oxaliplatin-induced liver toxicity (9–11), prompting the need for careful consideration of liver function due to the heightened risk of postoperative complications. Recent years have witnessed an increase in reported cases of portal hypertension linked to oxaliplatin-induced liver sinusoidal endothelial cell damage and obstruction (12–14). Nevertheless, it is imperative to distinguish this from the more prevalent complication arising from oxaliplatin liver damage known as SOS, with an incidence of 19 to 52% (15, 16). SOS is characterized by obstructive small vein thrombosis in hepatic endoscopic varices and presents with symptoms such as jaundice, right upper quadrant pain, hepatomegaly, ascites, and unexplained weight gain. In this particular case, the patient was referred due to portal hypertension, which was associated primarily with esophageal and gastric varices, splenomegaly, and a decreased platelet count. Notably, no clinical manifestations such as jaundice, abdominal pain, or ascites were observed. Pathological findings indicated vague nodular regenerative nodule formation, partial hepatocyte atrophy, and reduced mesenteric veins in portal areas resembling fractures. Characteristic pathological features of SOS, such as sinusoidal hemorrhage, thrombosis, or obstruction, were notably absent. In light of these findings, the patient was diagnosed with INCPH induced by oxaliplatin treatment. Some studies suggest that chemotherapy-induced liver toxicity may be dependent on both the duration and dosage of treatment. Nakano et al. (11) reported an increased risk of sinusoidal dilation with six or more cycles of oxaliplatin, and Slade et al. (17) reported that doses of oxaliplatin (85 mg/m^2^ every 2 weeks) for 10 or more cycles could induce sinusoidal portal hypertension and non-cirrhotic portal hypertension. In patients without a known history of liver disease who suddenly present with ascites, splenomegaly, esophageal varices, or thrombocytopenia (without other abnormalities in blood cells not attributable to post-chemotherapy myelotoxicity), oxaliplatin-induced portal hypertension should be suspected (18). Based on the detailed records of treatment regimens in the literature, as well as the 30 cases in this study, it is evident that if oxaliplatin is calculated strictly based on a dosage of 85 mg/m^2^, adjusted for ≥6 cycles, the proportion of patients is 86.7% (26/30), and for ≥10 cycles, the proportion is 66.7% (20/30). This suggests a trend that with longer cumulative treatment and higher doses, the likelihood of complications increases.

Concerning the etiology of oxaliplatin-induced portal hypertension, there is a proposition suggesting that the administration of oxaliplatin could potentially induce injury to sinusoidal endothelial cells. This, in turn, may give rise to the formation of intercellular gaps, resulting in cell detachment and subsequent obstruction (19, 20). Additionally, it has been reported that depletion of glutathione, consumption of nitric oxide, increased expression of matrix metalloproteinases in sinusoidal endothelial cells, and increased levels of vascular endothelial growth factors and activation of coagulation factors are involved in the pathogenesis of sinusoidal and portal venous diseases (21). A retrospective study found a direct correlation between the increase in spleen size and cumulative oxaliplatin use. In this study, the normalization of spleen size occurred rapidly, with 87% of patients showing a normal spleen size after 1 year and 100% after 1.5 years (22). However, another retrospective study found that 62.4% of patients exhibited an increase in spleen size during oxaliplatin-based chemotherapy, with an average increase of 18.7 ± 6.9% (range: 10–45.5%). Moreover, it is noteworthy that not all patients witnessed a complete return to normal spleen sizes by the conclusion of the study. The radiological indications of portal hypertension persisted and exhibited progression in 1.4% of cases (five patients), with ongoing deterioration observed in portal hypertension through imaging after treatment (23). The prognosis of the patient in this study aligns with the descriptions in this article. Even after the completion of oxaliplatin treatment, the degree of portal hypertension in this patient continued to increase, as evidenced by regular endoscopic examinations showing progressive variceal development. When reviewing the literature and taking into account 27 patients with detailed prognosis descriptions, it was found that 10 patients encountered a progressive exacerbation of portal hypertension and related complications. In parallel, seven patients exhibited diverse degrees of tumor progression, and six patients achieved favorable outcomes post-treatment. Additionally, the prognosis of six patients remains undetermined as they are still under ongoing follow-up.

INCPH is a rare condition characterized by portal hypertension in the absence of cirrhosis or other known causes of liver disease and visceral venous thrombosis. The etiological factors proposed in the current research can be broadly categorized into five classes: infection, immune disorders, predisposition to thrombosis, genetic factors, and a history of exposure to drugs or toxins (6). The pathology of INCPH primarily involves medium-sized fibrotic and dilated portal vein branches, often closely approaching or near the liver capsule. Subcapsular liver atrophy is observed, along with thickening and dilation of the main portal vein branches. Microscopically, fibrosis occurs in the portal vein branches, displaying a circular expansion. Some cases may exhibit incomplete fibrous septa formation, and the condition of peripheral portal vein narrowing, collapse, and branch absence is referred to as occlusive portal venopathy (24). Abnormally dilated thin-walled vessels are often found within the hepatic parenchyma near the portal vein branches. Proliferative nodules formed due to hepatocellular proliferation, regenerative nodular hyperplasia, and focal nodular hyperplasia-like lesions may also appear, possibly representing compensatory changes after hepatic parenchymal atrophy. Studies indicate that venous sclerosis, regenerative nodules, and residual portal vein branches are typical features of INCPH, along with sinusoidal dilation, the formation of periportal shunt vessels, and increased blood vessels within the hepatic parenchyma (25). Based on the current literature review, 24 patients underwent detailed liver tissue pathology examination, which primarily revealed sinusoidal dilation, congestion, portal vein occlusion, absence, narrowing, and shunting.

There are currently no randomized controlled trials for the prevention and treatment of variceal hemorrhage in INCPH patients, and there are no clear guidelines for their treatment. Therefore, the selection of treatment strategies mainly follows the guidelines for cirrhosis without any special treatment methods (5, 6, 26). However, a study by Siramolpiwat et al. (27) found that applying the treatment strategy based on the cirrhosis treatment protocol to INCPH patients can lead to good long-term outcomes. In general, discontinuing medications that can cause INCPH, using NSBBs, and endoscopic band ligation can be used for primary and secondary prevention of variceal hemorrhage. The main focus of INCPH treatment is on managing complications related to INCPH, such as ruptured esophagogastric varices, splenomegaly, splenic hyperfunction, and ascites. A total of 21 cases were treated according to literature summaries, with the primary emphasis on the treatment of ruptured esophagogastric varices. Among them, five cases achieved a favorable prognosis through ligation and/or sclerosing agent treatment, highlighting that the treatment approach primarily addresses complications, complemented by close follow-up and conservative medical management.

In conclusion, non-cirrhotic portal hypertension is a clinical consequence associated with oxaliplatin-induced liver injury, which may occur during treatment and persist for several years afterward. This condition has the potential to complicate subsequent therapies. Therefore, it is imperative to conduct routine monitoring of platelet levels and imaging, including invasive procedures such as endoscopy, in patients undergoing oxaliplatin treatment to identify any ongoing damage resulting from chemotherapy. Conservative medical management, when required, can be utilized to decelerate the progression of the disease. Alternatively, endoscopic ligation or sclerotherapy may be considered for high-risk lesions. Additionally, the possibility of INCPH should be considered in patients undergoing oxaliplatin treatment who experience persistent thrombocytopenia, varices, or ascites without other apparent causes.

## Data availability statement

The original contributions presented in the study are included in the article/Supplementary material, further inquiries can be directed to the corresponding author.

## Ethics statement

The studies involving humans were approved by Shangyu People's Hospital of Shaoxing Ethics Approval. The studies were conducted in accordance with the local legislation and institutional requirements. The participants provided their written informed consent to participate in this study. Written informed consent was obtained from the individual(s) for the publication of any potentially identifiable images or data included in this article.

## Author contributions

JY: Conceptualization, Data curation, Formal analysis, Writing – original draft, Writing – review & editing. YXi: Project administration, Resources, Supervision, Writing – review & editing. YXu: Project administration, Resources, Supervision, Writing – review & editing. NC: Data curation, Formal analysis, Writing – review & editing. YT: Data curation, Formal analysis, Writing – review & editing.
