# Supplementary material for: Case report: Oxaliplatin-induced idiopathic non-cirrhotic portal hypertension: a case report and literature review
Source: Front Med (Lausanne). 2023 Nov 28;10:1285064. doi: 10.3389/fmed.2023.1285064 (PMC10713788; doi:10.3389/fmed.2023.1285064)
Supplement: Supplementary file 1 [file Table_1.DOCX]

| Case number | Age | Gender | Dose | Time to onset | Complications | Imaging | Liver Biopsy | Treatment | Prognosis |
| --- | --- | --- | --- | --- | --- | --- | --- | --- | --- |
| 1 (1) | 68 | F | 1220mg | None | Esophageal variceal | varicosity | None | Unknown | Unknown |
| 2 (1) | 64 | F | 1226mg | None | Esophageal variceal | varicosity | None | Unknown | Unknown |
| 3 (1) | 68 | M | 1240mg | None | Esophageal varices with bleeding、ascites | varicosity | None | Unknown | Unknown |
| 4 (1) | 53 | M | 1760mg | None | Esophageal variceal | varicosity | Mild sinusoidal dilatation, and focal perivenular and perisinusoidal fibrosis | Unknown | Unknown |
| 5 (1) | 59 | F | 1880mg | None | Esophageal varices with bleeding | varicosity | None | Unknown | Unknown |
| 6 (2) | 68 | F | 13cycles | 24months | Esophageal varices with bleeding、thrombocytopenia、portal hypertension | varicosity、portal hypertension、Splenomegaly | mild steatosis and mild portal inflammation. In most of the portal tracts, the portal veins appear narrowed or are difficult to recognize | balloon-occluded retrograde transvenous obliteration | Unknown |
| 7 (3) | 51 | M | 6cycles  1320mg | None | Esophageal varices with bleeding、hepatic encephalopathy | varicosity、portal hypertension、nodular regenerative hyperplasia | the liver has a disturbed architecture with nodular  appearance of liver parenchyma characteristic of  nodular regenerative hyperplasia. Areas with sinusoidal congestion are also present | endoscopic band ligation and conservative measures | Recovery |
| 8 (4) | 57 | M | 10cycles  1623mg | 6months | Esophageal variceal、Splenomegaly、thrombocytopenia、ascites | Splenomegaly、varicosity、ascites | None | Follow-up visit | hyperascites |
| 9 (4) | 37 | F | 11cycles  1592mg | 12months | Esophageal varices with bleeding、Splenomegaly、thrombocytopenia | varicosity、Splenomegaly、Umbilical vein recanalization | None | Follow-up visit | Follow-up visit |
| 10 (4) | 59 | F | 12cycles  1740mg | 3months | Esophageal variceal、Splenomegaly、thrombocytopenia | varicosity、Splenomegaly、Umbilical vein recanalization | None | Unknown | Follow-up visit |
| 11 (4) | 39 | F | 12cycles  1752mg | 6months | Esophageal variceal、Splenomegaly、thrombocytopenia | varicosity、Splenomegaly、Umbilical vein recanalization | The liver parenchyma was noncirrhotic with narrowed/sclerotic portal veins, portal fibrosis, and mild nodular regenerative  hyperplasia, without significant portal inflammation. | partial hepatectomy | systemic chemotherapy |
| 12 (4) | 51 | M | 12cycles  2280mg | 7months | Esophageal varices with bleeding、ascites | Splenomegaly、varicosity、ascites、Collateral Circulation | None | endoscopic ligation and  propranolol | endoscopic ligation 3 times |
| 13 (4) | 69 | M | 15cycles  2491mg | 22months | Esophageal varices with bleeding、thrombocytopenia、portal hypertension、Splenomegaly | varicosity、Splenomegaly、Umbilical vein recanalization | None | endoscopic ligation and  conservative measures | Follow-up visit |
| 14 (5) | 45 | F | None | 9months | gastrointestinal hemorrhage | varicosity、portal hypertension | Focal Hepatocytic Necrosis/Dropout，Hepatoportal Sclerosis and Moderate Mixed Macrovesicular/Microvesicular Steatosis. Portal Tracts were Small，Most of the Portal Venules Appeared to be Absent/Obliterated | TIPS | Recovery |
| 15 (6) | 46 | F | None | 72months | gastrointestinal hemorrhage、Moderate esophageal varices， | Splenomegaly、varicosity | reticulin stains highlighting parenchymal nodularity and no significant fibrosis, suggestive of nodular regenerative hyperplasia. There was minimal steatosis and no significant portal or lobular inflammation | pantoprazole and nadolol | Follow-up visit |
| 16 (7) | 81 | F | 4cycles | None | Esophageal varices with bleeding、thrombocytopenia | varicosity | Features of nodular regenerative hyperplasia are better accentuated on reticulin stain, where linear zones of atrophic hepatocytes alternating with relatively hypertrophied hepatocytes are seen | cyanoacrylate glue therapy and conservative measures | Recovery |
| 17 (8) | 67 | M | 5cycles | 48months | Esophageal varices with bleeding | varicosity | Paraportal shunts,sinusoidal dilatation | Esophageal variceal bleeding treated by endoscopic ligation and beta-blockers | Persistence of small varices |
| 18 (8) | 47 | M | 6cycles | 8months | Splenomegaly、portal hypertension | Splenomegaly、portal hypertension、nodular regenerative hyperplasia | obliterative portal venopathy.paraportal shunts,nodular regenerative hyperplasia, sinusoidal dilatation | Follow-up visit | Development of large esophageal varices |
| 19 (8) | 78 | M | 6cycles | 36months | Esophageal varices with bleeding | varicosity | obliterative portal venopathy，paraportal shunts，  sinusoidal dilatation | Esophageal variceal bleeding treated by endoscopic ligation and beta-blockers | Recurrence of large varices re-treated with ligation and development of acute portal and splenic vein thrombosis |
| 20 (8) | 50 | F | 8cycles | 9months | Splenomegaly、portal hypertension | Splenomegaly、portal hypertension、Umbilical vein recanalization | Sinusoidal dilatation, obliterative portal venopathy,  paraportal shunts | Follow-up visit | persistence of splenomegaly |
| 21 (9) | 76 | M | 11cycles | 60months | Moderate esophageal varices、thrombocytopenia | Splenomegaly、varicosity、nodular regenerative hyperplasia | dilated sinusoids and collection of abnormal blood vessels around the hepatic central vein | Endoscopic injection sclerotherapy | hyperascites |
| 22 (10) | 63 | F | 37cycles | 36months | Esophageal varices with bleeding、thrombocytopenia | Splenomegaly、varicosity、nodular regenerative hyperplasia | The liver had a disturbed architecture with a nodular appearance in the liver parenchyma，Areas with sinusoidal congestion were also present.hardly  any areas of fibrous | endoscopic injection sclerotherapy | no further recurrence |
| 23 (11) | 69 | M | 4cycles  880mg | 3months | portal hypertension、gastrointestinal hemorrhage、Renal Failure | portal hypertension | central vein fibrosis with marked sinusoidal dilation and extravasation of red blood cells through the sinusoidal limiting plate. The hepatocytes show atrophic changes. | A transjugular intrahepatic portal-systemic shunt was placed. | Died |
| 24 (12) | 45 | M | 12cycles  1730mg | 6months | Splenomegaly、portal hypertension、thrombocytopenia | Splenomegaly、portal hypertension | The liver parenchyma showed diffuse sinusoidal dilatation and centrilobular vein fibrosis with liver cell necrosis without steatosis. | right hepatectomy | Follow-up visit |
| 25 (13) | 55 | F | 10cycles | 8months | gastrointestinal hemorrhage、Severe esophageal varices，thrombocytopenia | varicosity、portal hypertension、ascites | None | endoscopic ligation and conservative measures | Follow-up visit |
| 26 (14) | 78 | M | 10cycles | 42months | Splenomegaly、Esophageal variceal、portal hypertension、Collateral Circulation | Splenomegaly、varicosity、Collateral Circulation、portal hypertension | no evidence of hepatic parenchymal damage; sinusoidal dilatation was detected staining around the hepatic central vein area | endoscopic injection sclerotherapy plus endoscopic variceal ligation | Follow-up visit |
| 27 (15) | 63 | M | 13cycles | 24months | Esophageal varices with bleeding、Splenomegaly、ascites | Splenomegaly、portal hypertension、varicosity | None | endoscopic variceal ligation and TIPS andsplenectomize | hepatic encephalopathy，died |
| 28 (16) | 35 | F | 12cycles  1500mg | 15months | portal hypertension、Moderate esophageal varices，thrombocytopenia，Normal liver function | Splenomegaly、portal hypertension、varicosity | None | Follow-up visit | Unknown |
| 29 (17) | 68 | F | 14cycles  1248mg | None | portal hypertension、Splenomegaly、Mild Esophageal variceal | varicosity、portal hypertension、Splenomegaly、nodular regenerative hyperplasia | a nodular hepatic parenchyma without fibrosis，composed of large hyperplastic hepatocytes, were separated by strands of atrophic parenchyma showing compressed liver cell plates and sinusoidal congestion | Unknown | Died |
| 30 (17) | 55 | F | 18cycles  1715mg | None | portal hypertension、Splenomegaly、Mild Esophageal variceal | varicosity、portal hypertension、Splenomegaly、nodular regenerative hyperplasia | a nodular hepatic parenchyma without fibrosis，composed of large hyperplastic hepatocytes, were separated by strands of atrophic parenchyma showing compressed liver cell plates and sinusoidal congestion | Unknown | Died |
| 30 (17) | 71 | M | 6cycles  540mg | None | portal hypertension | portal hypertension、nodular regenerative hyperplasia | a nodular hepatic parenchyma without fibrosis，composed of large hyperplastic hepatocytes, were separated by strands of atrophic parenchyma showing compressed liver cell plates and sinusoidal congestion | Unknown | Died |
| 31 (18) | 61 | F | 3cycles | 48months | Esophageal variceal、Splenomegaly、thrombocytopenia | varicosity、portal hypertension、Collateral Circulation、Splenomegaly、nodular regenerative hyperplasia | no cirrhosis of the liver, bleeding around hepatic veins, loss of hepatic veins, and no dilation of sinusoids，loss of peripheral portal vein.a new paraportal shunting | endoscopic variceal ligation and transplantation | no further recurrence |
| 32 (19) | 64 | F | 8cycles | 16months | Esophageal varices with bleeding、Splenomegaly | Splenomegaly、portal hypertension | obvious sinusoidal dilatation，mild peri-sinusoidal fibrosis，obvious portal vein wall thickening and mild fibrosis in the portal area | proton pump inhibitor  and transfusion | no further recurrence |
| 33 (20) | 46 | M | 10cycles | 36months | portal hypertension、Severe esophageal varices，thrombocytopenia，Normal liver function | Splenomegaly、portal hypertension、varicosity、Collateral Circulation | Mild steatosis but without fibrosis or inflammation | endoscopic variceal ligation、venous embolism | Unknown |
| 34 (21) | 65 | M | 12cycles  1800mg | 15months | gastrointestinal hemorrhage，thrombocytopenia | Splenomegaly、varicosity、ascites | Unable to puncture due to excessive ascites | endoscopic injection sclerotherapy plus endoscopic variceal ligation | tumour progression |
| 35 (21) | 49 | F | 26cycles  3300mg | 30months | gastrointestinal hemorrhage，DIC，Hepatitis B activation，Normal liver function，thrombocytopenia | Splenomegaly、varicosity、Collateral Circulation、ascites | Unable to puncture due to excessive ascites | endoscopic injection sclerotherapy plus endoscopic variceal ligation | Unknown |

**REFERENCES**

1. Cha DI, Song KD, Ha SY, Hong JY, Hwang JA, Ko SE. Long-term follow-up of oxaliplatin-induced liver damage in patients with colorectal cancer. *Br J Radiol*. (2021) 94: 20210352. doi: 10.1259/bjr.20210352

2. Ramos Y, Gui D, Chak E. A rare cause of gastric variceal bleeding. *Case Rep Gastroenterol*. (2021) 15: 1008-12. doi: 10.1159/000521126

3. van den Broek MA, Olde DS, Driessen A, Dejong CH, Bemelmans MH. Nodular regenerative hyperplasia secondary to neoadjuvant chemotherapy for colorectal liver metastases. *Case Rep Med*. (2009) 2009: 457975. doi: 10.1155/2009/457975

4. Slade JH, Alattar ML, Fogelman DR, Overman MJ, Agarwal A, Maru DM, et al. Portal hypertension associated with oxaliplatin administration: Clinical manifestations of hepatic sinusoidal injury. *Clin Colorectal Cancer*. (2009) 8: 225-30. doi: 10.3816/CCC.2009.n.038

5. Lawal TO, Farris AB, El-Rayes BF, Subramanian RM, Kim HS. Oxaliplatin-induced hepatoportal sclerosis, portal hypertension, and variceal bleeding successfully treated with transjugular intrahepatic portosystemic shunt. *Clin Colorectal Cancer*. (2012) 11: 224-7. doi: 10.1016/j.clcc.2012.02.002

6. Ganta N, Prasad A, Aknouk M, Ghodasara K, Nair A, Taqvi Z, et al. A case report of nodular regenerative hyperplasia and non-cirrhotic portal hypertension post oxaliplatin chemotherapy. *Cureus*. (2022) 14: e28740. doi: 10.7759/cureus.28740

7. Tan YB, Teh J, Gwee YY, Ng YK. A review and case discussion on a rare cause of non-cirrhotic portal hypertension. *Cureus*. (2022) 14: e30252. doi: 10.7759/cureus.30252

8. Gioia S, Di Martino M, Minozzi M, Nardelli S, Cortesi E, Riggio O. Incidence of portal hypertension in patients exposed to oxaliplatin. *Dig Liver Dis*. (2019) 51: 1348-50. doi: 10.1016/j.dld.2019.06.020

9. Yazaki T, Kawashima K, Ishimura N, Kataoka M, Fukunaga M, Hyakudomi R, et al. Oxaliplatin-related portal hypertension complicated with esophageal varices and refractory massive ascites. *Intern Med*. (2022) 61: 3225-31. doi: 10.2169/internalmedicine.9266-21

10. Takaya H, Kawaratani H, Nakanishi K, Takeyama S, Morioka C, Sawai M, et al. Development of nodular regenerative hyperplasia (NRH) with portal hypertension following the administration of oxaliplatin for the recurrence of colon cancer. *Intern Med*. (2015) 54: 383-7. doi: 10.2169/internalmedicine.54.2461

11. Tisman G, MacDonald D, Shindell N, Reece E, Patel P, Honda N, et al. Oxaliplatin toxicity masquerading as recurrent colon cancer. *J Clin Oncol*. (2004) 22: 3202-4. doi: 10.1200/JCO.2004.99.106

12. Kang GH, Moon HS, Lee ES, Kim SH, Sung JK, Lee BS, et al. A case of liver fibrosis with splenomegaly after oxaliplatin-based adjuvant chemotherapy for colon cancer. *J Korean Med Sci*. (2013) 28: 1835-8. doi: 10.3346/jkms.2013.28.12.1835

13. Fuentes-Lacouture MC, Barrera-Garavito EC, Gomez A, Mantilla W. Non-Cirrhotic portal hypertension in a patient with colonic carcinoma treated with oxaliplatin. *J Med Cases*. (2021) 12: 99-101. doi: 10.14740/jmc3630

14. Shigefuku R, Watanabe T, Mizukami T, Matsunaga K, Hattori N, Ehira T, et al. Esophagogastric varices were diagnosed in a non-cirrhotic liver case during long-term follow-up after oxaliplatin-based chemotherapy. *Clin J Gastroenterol*. (2018) 11: 487-92. doi: 10.1007/s12328-018-0873-1

15. Morioka D, Izumisawa Y, Yamaguchi K, Sato K, Komiyama S, Nakagawa K, et al. Surgical intervention for portal hypertension caused by oxaliplatin-based chemotherapy: A case report and a review of literature regarding radiological and/or surgical interventions for oxaliplatin-associated portal hypertension. *Clin J Gastroenterol*. (2020) 13: 799-805. doi: 10.1007/s12328-020-01157-w

16. Heo J, Shin KY, Kwon YH, Park SY, Jung MK, Cho CM, et al. [a case of portal hypertension after the treatment of oxaliplatin based adjuvant chemotherapy for rectal cancer]. *Korean J Gastroenterol*. (2011) 57: 253-7. doi: 10.4166/kjg.2011.57.4.253

17. Hubert C, Sempoux C, Horsmans Y, Rahier J, Humblet Y, Machiels JP, et al. Nodular regenerative hyperplasia: A deleterious consequence of chemotherapy for colorectal liver metastases? *Liver Int*. (2007) 27: 938-43. doi: 10.1111/j.1478-3231.2007.01511.x

18. Tanji Y, Furukawa K, Igarashi Y, Yanagaki M, Haruki K, Shirai Y, et al. Living donor liver transplantation for idiopathic portal hypertension with focal nodular hyperplasia. *Surg Case Rep*. (2022) 8: 73. doi: 10.1186/s40792-022-01428-3

19. Zhang X, Gao YY, Song DZ, Qian BX. Isolated gastric variceal bleeding related to non-cirrhotic portal hypertension following oxaliplatin-based chemotherapy: A case report. *World J Gastroenterol*. (2022) 28: 3524-31. doi: 10.3748/wjg.v28.i27.3524

20. 緒方大聡, 具嶋敏文, 丸岡新子, 高崎智子, 田中吏佐, 松浦隆志, et al. MFOLFOX6療法後に発症した門脈圧亢進症の1例. *日本消化器病学会雑誌*. (2013) 110: 2119-26. doi: 10.11405/nisshoshi.110.2119

21. 重福隆太, 高橋秀明, 津田享志, 池田裕喜, 松永光太郎, 松本伸行, et al. 大腸癌化学療法中に食道胃静脈瘤破裂をきたした2症例. *日本消化器病学会雑誌*. (2014) 111: 2326-36. doi: 10.11405/nisshoshi.111.2326
